# Deficits in Executive and Memory Processes in Delusional Disorder: A Case-Control Study

**DOI:** 10.1371/journal.pone.0067341

**Published:** 2013-07-02

**Authors:** Inmaculada Ibanez-Casas, Enrique De Portugal, Nieves Gonzalez, Kathryn A. McKenney, Josep M. Haro, Judith Usall, Miguel Perez-Garcia, Jorge A. Cervilla

**Affiliations:** 1 Centro de Investigacion Biomedica en Red de Salud Mental (CIBERSAM) University of Granada, Granada, Spain; 2 Federico Olóriz Institute of Neurosciences, University of Granada, Granada, Spain; 3 Psiquiatria Biologico Ambiental (PSYBAM Group),University of Granada, Granada, Spain; 4 Department of Psychiatry, Gregorio Marañon Hospital, Madrid, Spain; 5 Research and Development Unit, Sant Joan de Déu-SSM, Parc Sanitari Sant Joan de Déu, C/Doctor Antoni Pujadas, 42, 08830- Sant Boi de Llobregat, Barcelona, Spain; 6 Centro de Investigacion Biomedica en Red de Salud Mental (CIBERSAM) San Juan de Dios Foundation, Barcelona, Spain; 7 Department of Personality, Assessment and Psychological Treatment, University of Granada, Granada, Spain; 8 Department of Psychiatry and Medical Psychology, University of Granada, Granada, Spain; 9 Psychiatric Inpatient Unit, San Cecilio University Hospital, Granada, Spain; 10 Centre for Global Mental Health, Health Services and Population Research Department, Institute of Psychiatry, King's College London, London, United Kingdom; Maastricht University Medical Centre, The Netherlands

## Abstract

**Objective:**

Delusional disorder has been traditionally considered a psychotic syndrome that does not evolve to cognitive deterioration. However, to date, very little empirical research has been done to explore cognitive executive components and memory processes in Delusional Disorder patients. This study will investigate whether patients with delusional disorder are intact in both executive function components (such as flexibility, impulsivity and updating components) and memory processes (such as immediate, short term and long term recall, learning and recognition).

**Methods:**

A large sample of patients with delusional disorder (n = 86) and a group of healthy controls (n = 343) were compared with regard to their performance in a broad battery of neuropsychological tests including Trail Making Test, Wisconsin Card Sorting Test, Colour-Word Stroop Test, and Complutense Verbal Learning Test (*TAVEC*).

**Results:**

When compared to controls, cases of delusional disorder showed a significantly poorer performance in most cognitive tests. Thus, we demonstrate deficits in flexibility, impulsivity and updating components of executive functions as well as in memory processes. These findings held significant after taking into account sex, age, educational level and premorbid IQ.

**Conclusions:**

Our results do not support the traditional notion of patients with delusional disorder being cognitively intact.

## Introduction

Delusional disorder (DD) is a psychotic disorder whose most prominent symptomatology is the presence of delusional beliefs [1]. The disorder is, thus, characterized by false beliefs that are held with high certainty despite evidence against them and that are typically accompanied by negative affect and exaggerated vigilance [2]. These delusional beliefs are usually monothematic and encapsulated, and normally lack the bizarreness of schizophrenic delusions [3].

Many authors have proposed that patients with DD are cognitively intact, and some have even suggested that this would be a prerequisite for diagnosis, since elaborated delusions require an intact neurocognitive system [2]. Besides, one of the clinically defined features of DD is precisely the lack of a marked functional impairment [4]. However, very limited evidence on executive functiońs components and memory processes in DD patients exists to date, even though neuropsychological research might provide support to some of the current theories accounting for delusion formation and maintenance in DD [5].

On the other hand, recent studies have demonstrated that executive function is far from being a unitary concept [6], [7]. Three of the most postulated executive functiońs components proposed in the literature are *Flexibility* or *Shifting* (Ability to shift between different tasks or elements of the same task), *Impulsivity* or *Inhibition* (ability to inhibit inappropriate responses) and *Updating* (ability to incorporate relevant information and remove non relevant). Although they seem to correlate with each other, it has also been proven that they are clearly distinct components of executive function [8]–[11]. We find it reasonable to hypothesize that impairments in these components could be related with delusional symptoms in DD. Thus, a failure in *Flexibility* could account for the rigidity with which DD patients hold their delusional beliefs. Similarly, some known cognitive biases of psychosis, such as an inability to ignore implausible explanations or a tendency to early jumps to conclusions (JTC), could be based on executive function impairments such as *Impulsivity* or *Inhibition*. Finally, malfunction at the *Updating* component could cause difficulties in managing information what, in turn, could prompt delusional explanations. Besides, the role of memory processes in the formation and maintenance of delusions remains unclear, although they could also be at the basis of some of biases consistently reported to lead to delusion formation.

Given the split nature of executive functions and memory processes, separate neuropsychological measures are needed [12]. Only a handful of studies with limited samples sizes has investigated complex cognitive functions such as attention, verbal and motor skills, abstraction, cognitive flexibility, verbal and sustained attention and verbal learning and memory in patients with DD [13]–[22]. Some of these studies have shown subtle impairments in executive functions and memory among DD patients as compared to controls, and some others found little differences between DD patients and patients with paranoid schizophrenia, supporting the currently prevailing notion of a continuum of psychotic symptoms [23].

Despite the acknowledged importance of executive and memory processes in psychotic disorders, very little empirical research has been done to date to clarify the neuropsychological mechanisms underpinning DD. The present study aims to empirically explore memory and executive components of flexibility, impulsivity and updating in a relatively large sample of DD cases as compared with a large group of healthy controls. We hypothesized that DD patients will show impairments in executive function components and memory and learning processes as compared to healthy controls.

## Methods

### Ethics Statement

All participants were provided with a complete description of the study and returned a signed written informed consent prior to their voluntary participation. In patients legally deemed compromised in their decision-making, we sought additional signed agreement from those holding power of attorney. This study was approved by the ethics committees of Sant Joan de Déu-Mental Health Service (Barcelona) and University Hospital San Cecilio (Granada). This study has been performed in accordance with the ethical standards laid down in the 1964 Declaration of Helsinki.

### Participants

This is a case-control study in which 429 participants were included (86 DD cases and 343 healthy controls). DD cases sampling is described in detail elsewhere [24]. In brief, cases were obtained from the computerized case register of Sant Joan de Déu-Serveis de Salut Mental (San Joan de Déu-Mental Health Services, SJD-SSM). Inclusion criteria were: *a)* fulfilling diagnostic criteria for DD after administering the *Structured Clinical Interview for DSM-IV Axis I Disorders (SCID-I)* [25]; *b)* being older than 18 years of age; *c)* living in the catchment area of SJD-SSM; *d)* having attended the outpatient clinic at least once over the previous six months; and *e)* patient’s agreement to participate. Exclusion criteria were: *a)* not completely fulfilling diagnostic criteria for DD; and *b)* having a diagnosis of mental retardation. Of the total sample of patients with a diagnosis of DD in the computerized case register, 106 were randomly selected for this study. Of the 106 selected cases, 6 patients refused to participate in the study, 8 were not invited to take part by their psychiatrist, and six patients did not have a confirmed diagnosis of DD. Thus, the final sample for the study consisted of 86 DD cases. A summary of their main clinical features is provided in Table 1.

**Table 1:** Sociodemographic and clinical descriptives of patients with delusional disorder (n = 86).

Sex	n (%)
Male	33 (38.4)
Female	53 (61.6)
Subtype of delusional disorder	
Persecutory	51 (59.3)
Jealous	19 (22.1)
Somatic	3 (3.5)
Erotomaniac	4 (4.7)
Grandiose	4 (4.7)
Mixed	5 (5.8)
	Mean± SD
Age at assessment *(Years)*	53.9±14.4
Age at onset *(Years)*	39.6±14.3
Years since onset	14.6±12.2
Psychopathology	
PANSS positive scale	13.8±4.5
PANSS negative scale	9.9±2.8
PANSS generalpsychopathology scale	23.8±4.8
Functionality	
Cognitive (MMSE)	27.6±2.4
Social (GAF)	63.9±11.3

**Key**: PANSS, Positive and Negative Syndrome Scale for Schizophrenia; MMSE, Mini Mental State Examination; GAF, Global Assessment of Functioning.

On the other hand, a total of 352 controls volunteered to participate in this study. They were all recruited in Granada (Spain) while attending a training program for unemployed people organized by the Andalusian Employment Service. Inclusion criteria were: *a)* Not fulfilling diagnostic criteria for any current psychotic, severe affective disorder (mania or severe depression) or substance related disorder DSM-IV Axis I as identified after administering the *Mini International Neuropsychiatric Schedule (M.I.N.I.)* [26] (9 participants excluded); *b)* being older than 18 years of age. Exclusion criteria were: a*)* scores on MEC-30 under 24; *b*) overt traumatic brain injury; *c)* significant loss of consciousness; and *d)* neurological disorder. A final sample of 343 healthy participants took part in this study. Not all controls undertook all tests due to the multiple assessment sessions, and Table 2 shows the groupś sizes for each measure.

**Table 2:** Sociodemographic data and comparisons between patients with delusional disorder and healthy control groups.

	Cases(n = 86)	Control Group forTrail Making Test(n = 336)			Control Group forWisconsin Card Sorting Test(n = 31)			Control Group forColor-Word Stroop Test(n = 75)			Control Group forTAVEC Test(n = 267)		
	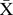 (SD)	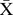 (SD)	F	P	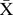 (SD)	F	p	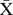 (SD)	F	p	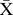 (SD)	F	p
**Age** *(Years)*	53.97(14.43)	34.90(11.11)	163.30	<.05*	30.58(10.57)	68.03	<.05*	25.97(3.84)	265.44	<.05*	37.86(11.42)	112.99	.<.05*
**Educational Level** *(Years)*	8.77(5.09)	12.18(4.21)	37.50	<.05*	14.29(4.07)	29.56	<.05*	13.00(4.11)	33.02	<.05*	11.77(4.38)	28.15	<.05*
**Premorbid IQ**	115.00(14.13)	118.11(13.10)	4.74	.30*	121.38(12.59)	4.88	.029*	118.07(20.28)	6.41	.012*	117.61(13.21)	2.85	.04*
	**No. Women** **(%)**	**No. Women** **(%)**			**No. Women** **(%)**			**No. Women** **(%)**			**No. Women** **(%)**		
**Sex**	*53* *(61.6)*	*178* *(53.0)*		.492†	*24* *(77)*		.112†	*37* *(49.3)*		.152†	*122* *(45.7)*		.010* †

† Chi-square asymptotic significance (bilateral).

### Instruments and Measures

The interviews and neuropsychological assessment took approximately two hours in healthy controls and four hours in DD cases. The difference in time between patients and controls was mainly due to the clinical assessment in patients that was not performed in healthy controls, and to the increased time taken by patients to perform the tasks. To avoid bias related to fatigue, the assessment was distributed in different sessions. Participants were assessed by post-graduate psychologists who were specifically trained to administer all used instruments.

#### Sociodemographical and clinical assessment

The sociodemographic factors recorded for this study were: a) Sex: male/female; b) Age: in years; c) Educational level: in years of schooling; and d) premorbid IQ: estimated by a Spanish version of Barona, Reynolds, & Chastiańs [27] formula developed by Bilbao-Bilbao and Seisdedos [28].

#### Neuropsychological assessment: Flexibility domain

*Trail Making Test (TMT)* [29]. The TMT has been described as a visuomotor tracking task with different processing demands for each part [30]. Some studies have shown that particularly TMT B is an excellent measure of *Shifting* (flexibility) [31], [32].

Computerized version of *Wisconsin Card Sorting Test (WCST)* [33]. The WCST is one of the most frequently used tests assessing executive functioning in clinical settings [34]. WCST is generally used to measure the capacity to deduce concepts and to apply a strategy to adapt behaviour to changing conditions [30]. Recent studies have shown that errors primarily tap on the *Shifting/Flexibility* component of executive function [8]–[11].

Both TMT and WCST have recently been shown to be independent from psychotic positive symptoms severity [35], which makes them valuable tools when assessing both patients and healthy controls.

#### Neuropsychological assessment: Impulsivity domain

*Colour-Word Stroop Test (STROOP)* [*36*]. The Colour-Word Stroop Test measures cognitive processing and dimensions such as cognitive flexibility, resistance to interference from outside stimuli and creativity [30]. Colour-Word Stroop test has been proved to tap primarily on the *Inhibition/Impulsivity* component of executive functioning [11], .

#### Neuropsychological assessment: Updating domain (reasoning and working memory)

This domain was assessed using the number of categories completed in the WCST and the score in immediate recall in trial 1 on the TAVEC test. The former is considered to be a sensitive indicator of *abstract reasoning* [38] which is a key element for the *Updating* component of executive function [11].

The latter has been shown to be a sensitive indicator of *working memory span* [30], hence also assessing the *Updating* component of executive function.

#### Neuropsychological assessment: Memory and learning domain

*Complutense Verbal Learning Test (TAVEC)* [39]. The TAVEC is the Spanish version of the California Verbal Learning Test (CVLT) [40], [41] and is used for the assessment of different memory and learning processes, such as immediate recall, short and long term memory with and without semantic clues and recognition.

### Statistical Analyses

First, an analysis to check whether cases and controls were equivalent on the main socio-demographic variables (sex distribution, age and educational level) was performed. Premorbid IQ was also included given that it is known to be a relevant variable regarding neuropsychological tests variance. Since not all controls undertook the full battery of tests, these analyses were performed on a group by group basis. Sex distribution differences were analyzed using Chi-square tests, and the remaining demographic factors were explored using univariate ANOVA tests. Differences on the neuropsychological tests scores between cases and controls were investigated via univariate ANCOVA analyses in order to reduce the potential confounding effect of sex distribution, age, years of schooling and premorbid IQ.

We used Bonferroni method to correct for multiple testing and established a p-value of.002 to consider a difference as statistically significant.

## Results

The sociodemographic data for all the groups participating in this study are shown in Table 2. DD cases showed to be older and to have a lower educational level and a lower premorbid IQ than controls. On the contrary, no statistically significant difference was found between cases and controls in sex distribution except for TAVEC test group, with a higher percentage of women among the cases. Given that group size for WCST was the shortest, analyses comparing WCST group versus the remaining groups in terms of sex distribution, age, educational level and premorbid IQ were also performed. These showed statistically significant differences for all the studied sociodemographic variables (all p<.05). All these results justified the use of ANCOVA analyses, controlling for sex distribution (where necessary), age, educational level and premorbid IQ.

Unadjusted and adjusted comparisons between DD cases and controls in the four studied domains are shown in detail in Tables 3 to 6.

**Table 3:** Descriptive scores and comparisons between patients with delusional disorder and healthy controls in the flexibility domain.

	Calculated Meansand Standard Deviations		Unadjusted Model		Estimated Marginal Meansand Standard Error		95% Confidence Interval		Adjusted Model		Coheńs d
	Cases	Controls			Cases	Controls	Cases	Controls	(by age, educational level and premorbid IQ)		
**TMT**	(n = 86)	(n = 336)	**F**	**p**	(n = 86)	(n = 336)	(n = 86)	(n = 336)	**F**	**p**	
Part A (secs.)	105.33 (71.26)	38.32 (16.97)	105.78	**<.002** *	86.18 (4.14)	42.42 (1.70)	78.04–94.32	39.07–45.78	86.98	**<.002** *	1.52
Part B (secs.)	259.30 (237.89)	87.57 (42.06)	67.09	**<.002** *	209.35 (12.92)	97.53 (5.16)	183.95–234.75	87.38–107.68	59.51	**<.002** *	1.23
**WCST**	(n = 86)	(n = 31)	**F**	**p**	(n = 86)	(n = 31)	(n = 86)	(n = 31)	**F**	**p**	
Total Errors	28.65 (11.17)	35.84 (20.04)	5.23	**.024** *	26.64 (1.82)	40.23 (3.02)	23.01–30.27	34.24–46.23	11.97	**.001** *	.46
Perseverant Responses	16.63 (11.44)	19.81 (10.64)	1.71	.194	15.22 (1.47)	22.90 (2.43)	12.29–18.15	18.07–27.73	5.88	.017	.29
Perseverant Errors	14.16 (8.62)	17.97 (9.25)	3.96	**.049** *	13.11 (1.16)	20.26 (1.92)	10.80–15.42	16.45–24.08	8.18	.005	.43
Non-Perseverant Errors	14.49 (9.04)	17.87 (11.65)	2.48	.119	13.52 (1.28)	19.97 (2.12)	10.97–16.08	15.75–24.19	5.43	.022	.33
Conceptual Level Responses	26.52 (15.38)	63.23 (15.98)	119.51	**<.002** *	29.05 (1.84)	57.65 (3.04)	25.39–32.71	51.62–63.69	52.24	**<.002** *	2.34
No. of Categories	1.68 (1.4)	4.61 (1.85)	70.60	**<.002** *	2.05 (.19)	3.78 (.31)	1.67–2.43	3.15–4.41	17.48	**<.002** *	1.80

* Level of significance p = .002 after Bonferroni correction.

**Table 4:** Descriptive scores and comparisons between patients with delusional disorder and healthy controls in the inhibition/impulsivity domain.

	Calculated Meansand Standard Deviations		Unadjusted Model		Estimated Marginal Meansand Standard Error		95% Confidence Interval		Adjusted Model		Coheńs d
	Cases	Controls			Cases	Controls	Cases	Controls	(by age, educational level and premorbid IQ)		
**STROOP TEST**	(n = 86)	(n = 75)	**F**	**p**	(n = 86)	(n = 75)	(n = 86)	(n = 75)	**F**	**p**	
Words	78.46 (22.60)	98.63 (5.45)	56.00	**<.002** *	88.01 (2.50)	90.34 (2.24)	83.06–92.96	85.89–94.79	22.94	**<.002** *	1.44
Colours	54.14 (16.86)	75.97 (11.09)	84.35	**<.002** *	63.58 (2.08)	67.78 (1.87)	59.46–67.70	64.07–71.49	37.23	**<.002** *	1.56
Words & Colours	34.05 (12.50)	46.91 (11.57)	39.88	**<.002** *	37.84 (1.97)	43.61 (1.77)	33.93–41.75	40.09–47.13	14.91	**<.002** *	1.07
PĆ	31.89 (9.30)	42.71 (4.01)	85.80	**<.002** *	36.77 (1.01)	38.38 (.91)	34.77–38.77	36.58–40.18	40.64	**<.002** *	1.63
Interference	2.27 (8.50)	4.18 (9.99)	1.47	.228	1.07 (1.63)	5.22 (1.47)	−2.16–4.30	2.32–8.13	2.09	.070	.21

* Level of significance p = .002 AFTER Bonferroni correction.

**Table 5:** Descriptive scores and comparisons between patients with delusional disorder and healthy controls in the updating domain.

	Calculated Meansand Standard Deviations		Unadjusted Model		Estimated Marginal Meansand Standard Error		95% Confidence Interval		Adjusted Model		Coheńs d
	Cases	Controls	F	p	Cases	Controls	Cases	Controls	(by age, educational level and premorbid IQ)		
									F	p	
No. of Categories (WCST)	1.68 (1.4)	4.61 (1.85)	70.60	**<.002** *	2.05 (.19)	3.78 (.31)	1.67–2.43	3.15–4.41	17.48	**<.002** *	1.80
Immediate Recall Trial 1 (TAVEC)	4.77 (2.13)	6.47 (2.04)	32.63	**<.002** *	5.49 (.25)	6.27 (.12)	5.00–5.99	6.03–6.51	25.45	**<.002** *	.82

* Level of significance p = .002 after Bonferroni correction.

**Table 6:** Descriptive scores and comparisons between patients with delusional disorder and healthy controls in memory and learning.

	Calculated Meansand Standard Deviations		Unadjusted Model		Estimated Marginal Meansand Standard Error		95% Confidence Interval		Adjusted Model		Coheńs d
	Cases	Controls							(by sex distribution, age, educational level and premorbid IQ)		
**TAVEC**	(n = 86)	(n = 267)	**F**	**p**	**Cases**	**Controls**	**Cases**	**Controls**	**F**	**p**	
Immediate Recall Trial 1	4.77 (2.13)	6.47 (2.04)	32.63	**<.002** *	5.49 (.25)	6.27 (.12)	5.00–5.99	6.03–6.51	25.45	**<.002** *	.82
Immediate Recall Trial 5	10.24 (3.26)	12.79 (2.77)	19.55	**<.002** *	11.17 (.36)	12.53 (.17)	10.45–11.89	12.18–12.87	19.82	**<.002** *	.85
Total Words	41.31 (12.97)	53.06 (9.31)	19.12	**<.002** *	46.11 (1.20)	51.72 (.58)	43.74–48.49	50.57–52.87	43.88	**<.002** *	1.05
Short Term Free Recall	8.15 (3.76)	11.65 (2.79)	17.63	**<.002** *	9.50 (.36)	11.27 (.17)	8.79–10.22	10.93–11.62	40.77	**<.002** *	1.07
Long Term Free Recall	8.68 (3.81)	12.19 (2.78)	22.19	**<.002** *	10.04 (.36)	11.80 (.17)	9.32–10.76	11.46–12.15	40.63	**<.002** *	1.07
Perseverations	7.95 (6.20)	5.69 (4.89)	8.55	**<.002** *	7.52 (.68)	5.81 (.33)	6.18–8.87	5.15–6.46	3.33	**.011**	.41
Intrusions in Free Recall	4.96 (4.38)	3.13 (3.18)	7.10	**<.002** *	4.38 (.44)	3.28 (.21)	3.50–5.26	2.86–3.71	8.225	**<.002** *	.48
Hits in Recognition	13.38 (2.67)	15.12 (1.16)	10.17	**<.002** *	13.59 (.21)	15.06 (.10)	13.18–14.01	14.86–15.26	18.63	**<.002** *	.91
False Positives in Recognition	3.30 (3.83)	1.42 (3.16)	3.64	.059	2.45 (.29)	1.47 (.14)	1.88–3.02	1.19–1.74	25.34	**<.002** *	.54

* Level of significance p = .002 after Bonferroni correction.

When the *Flexibility* domain was considered (Table 3), results showed statistically significant differences both in part A and B of the Trail Making Test, cases being slower than controls. Very large effect sizes were found for both measures. Analyses also showed statistically significant differences on all WCST variables with the initial level of significance (p<0.05), but significance disappeared for *Perseverant Responses*, *Perseverant Errors* and *Non-Perseverant Errors* scores when the corrected level of significance was adopted (p<0.002). Cases tended to make significantly fewer *Total* Errors showing a small effect size of.46. Besides, the number of *Conceptual Level Responses* and *No. of categories* were also significantly smaller in cases than in healthy controls and effect sizes for both variables were very large.

In the *Impulsivity* domain (Table 4), the differences between cases and controls on the majority of scores were statistically significant and effect sizes ranged from 1.07 to 1.63. Cases tended to name fewer *Words* and fewer *Colours*, to show lower scores on the *Word & Colour* subtest, and to have a lower *PC’* index. However, scores in *Interference* were not statistically different for cases and controls.

As for the *Updating* domain (Table 5), results showed that the N*umber of Categories* completed was significantly smaller in cases than in controls and effect size was very large (Coheńs d of 1.80). Also, cases showed a significantly poorer performance on *Immediate Recall in Trial 1, with a large effect size of.82*.

Finally, with regard to memory and learning processes (Table 6), statistically significant differences were found in all the studied variables except for *Perseverations*. Cases showed lower scores on *Immediate Recall at trial 1*, *Immediate Recall at trial 5*, *Total Words*, *Short* and *Long Term Free Recall* and *Hits in Recognition.* Additionally, cases showed higher scores on most error variables in this test, this is: *Intrusions in Free Recall* and *False Positives in Recognition*. Effect sizes were large or very large for all the scores except for *Intrusions in Free Recall* and *False Positives in Recognition* (Coheńs d of.41 and.54 respectively).

## Discussion

### Main Findings, Strengths and Limitations

In summary, our results show empirical evidence that DD cases performed worse than controls in both a variety of components of executive function and in memory tasks. Thus, DD patients showed lower levels of flexibility, slower speed processing, restricted capacity for learning, updating and inhibiting inappropriate information and poorer memory and reasoning. Our findings were independent of potential confounding effects of sex, age, educational level and premorbid IQ.

Some authors have postulated that patients with DD are cognitively intact [2] and this disorder has indeed been traditionally described as not evolving to defective states [4], [42]. However, our findings provide consistent empirical evidence demonstrating the contrary, at least on the tested areas of executive function, memory and learning. It must be said, though, that a few studies, using smaller samples and without the direct objective of looking into these specific cognitive functions, had previously provided some similar evidence [13], [14], [43]. However, to the best of our knowledge, this is the first study investigating all components of executive function and memory processes together in a relatively large sample of DD patients as compared to healthy controls.

The main advantage of the study is the relatively large sample of 86 rare DD cases. However, a number of limitations do exist. Given that cases come from a clinical outpatient setting, controls were necessarily obtained from a different source. Indeed, controls were drawn from a selected pool of unemployed people and come from a different geographical province in Spain and this can limit the generalizability of our findings. Admittedly, a degree of selection bias was present as cases and controls tended to differ systematically in most sociodemographic variables. Nonetheless, in an attempt to counteract such plausible biases, we performed ANCOVA analyses adjusting for all the potential confounders, including sociodemographic and educational variables. Additionally, a high attrition rate was observed in the control group. This happened mainly because participants finished or quitted the course they were attending and/or they moved to another city trying to find a job. Lack of detailed personal information due to confidentiality concerns and difficulties in providing participants with appropriate incentives also contributed to this attrition rates. However, analyses comparing those who dropped out with participants who completed both sessions showed no significant difference (all p>.05) in most sociodemographic measures, meaning there was no attrition bias in our control sample.

### Flexibility

As pointed out by Abdel-Hamid *& Brüne* [3], cognitive flexibility in DD patients had not been previously tested. In our study, we found that DD cases had a poorer performance in most *Flexibility* tasks. For instance, cases were slower in TMT part B and effect size for this variable was very large (Coheńs d = 1.23), which is generally interpreted as poor cognitive flexibility [44]. Furthermore, WCST has classically been one of the preferred tests to measure cognitive flexibility. In our study, DD cases unexpectedly showed better scores (fewer errors) on *Total Errors* although effect size showed to be quite small (Coheńs d = .46). Previous findings in patients with DD [14] and other psychotic patients [45] made it reasonable to expect the opposite finding. Nevertheless, our findings might be explained by the hyper-attentiveness consistently found in DD patients [46]. This high vigilance together with the motivational need to avoid harm (errors) could be a plausible explanation for our results, and would give support to theories based on perceptive and motivational deficits in DD [5]. On the other hand, patients showed a significantly lower number of C*onceptual Level Responses* and *Categories* with very high effect sizes (Coheńs d of 2.34 and 1.80 respectively), both measures being considered as prime elements of the flexibility component within WCST. On the whole, our interpretation is that the *Flexibility* component of executive function is impaired in patients with DD.

### Impulsivity

Performance in the Stroop test reflects functioning on the *Inhibition/Impulsivity* domain. The number of *Words* and of *Colours* was significantly smaller among cases, which is an evidence for low processing speed. Although Stroop tests have been traditionally considered a measure of focused attention and processing speed, they also assess inhibition of inappropriate responses since they point to the ability to change attention voluntarily from one aspect of the stimulus to another [47]. Scores in Interference showed to be not statistically different between cases and controls in our sample. However, cases did tend to show a smaller number of colours named in the W*ord & Colour* task with a very large effect size of 1.07, which points to the existence of an impairment in *Inhibition* among DD patients.

### Reasoning and Working Memory

Cases completed a lower *Number of Categories* in the WCST. This score is highly related to *abstract reasoning*, which is one of the most popular factors contributing to the *Updating* domain. Besides, our results showed that DD cases scored significantly below controls on *Immediate Recall in Trial 1*, indicating a reduced working memory span. Besides, effect sizes for both measures were quite large (Coheńs d of 1.80 and.82 respectively). Taking all these results together we conclude that this impairment in the *Updating* domain might make it difficult to manage information and interact with the social environment which could, in turn, contribute to delusion formation (47).

### Memory and Learning

In addition to lower scores in *Immediate Recall in Trial 1*, cases also showed lower scores in *Immediate Recall in Trial 5* and *Total Words,* suggesting that DD cases have also difficulties in learning new information. Moreover, when we compared immediate and delayed free recall (*Short Term* and *Long Term Free Recall*) with *Recognition*, we found higher scores on the latter, which is a characteristic pattern of impairment in retrieval memory. Unexpectedly, cases and controls did not differ in *Perseverations* what, again, might point to hypervigilance, a trait that can be found in patients with DD. Furthermore, cases scored higher than controls on *False Positives in Recognition,* suggesting that indeed these patients could be highly motivated to avoid harm (i.e., errors) and hyperattentive [3]. However, this increased number of false positives was not associated with a higher score in *Recognition Hit*, an indication of a potential general impairment in long term memory plus a bias toward making up for their deficit via false positives recognition. In summary, DD cases showed a poorer performance on all memory tasks, including immediate, short and long term memory with large to very large effect sizes, which might be against Garety & Freemańs [48] notion that DD patientś Jumping to Conclusions (JTC) bias is not a consequence of a memory deficit. We suggest that future research should address the role of memory deficits in this and other cognitive biases found in psychoses.

### Conclusion

We demonstrated that executive functions components such as *Flexibility*, *Impulsivity, reasoning and working memory* together with memory and learning processes are clearly poorer in DD cases than in healthy controls. This is true with the exception of some areas on which cases seem to perform better than controls, possibly indicating hypervigilance to some selectively abstracted stimuli [49]. Our results are not in line with the traditional notion of cognitive preservation in DD patients. On the contrary, if compared with previous studies on schizophrenia, executive function in DD could be postulated as being half-way between that of patients with schizophrenia and controls [50]. However, our sample of cases and controls differ in some essential characteristics and the generalizability of our findings is limited as our results need replication from more robust studies comparing DD cases with matched controls from the same population pool.

## References

[1] Munro A. (1999) Delusional disorder: Paranoia and related illnesses. New York: Cambridge University Press.

[2] Kunert HJ, Norra C, Hoff P (2007) Theories of delusional disorders - An update and review. Psychopathology 40: 191–202. 17337940 10.1159/000100367

[3] Abdel-Hamid M, Brüne M (2008) Neuropsychological aspects of delusional disorder. Curr Psychiatr Rep 10: 229–234. 10.1007/s11920-008-0038-x 18652791

[4] American Psychiatric Association (2000) Diagnostic and Statistical Manual of Mental Disorders, 4th edn, text revision (DSM-IV-TR). Washington, D.C.

[5] Ibanez-Casas I, Cervilla J (2012) Neuropsychological research in delusional disorder: a comprehensive review. Psychopathology 45: 84–101. 10.1159/000327899 22269940

[6] Baddeley A (1996) Exploring the central executive. Q J Exp Psychol A 49: 5–28.

[7] Cummings JL, Royall RD, Reeve A, Rummans TA, Kaufer DI, et al. (2002) Executive Control Function: A review of its promise and challenges for clinical research. J Neuropsychiatry Clin Neurosci 377–405. 10.1176/jnp.14.4.377 12426407

[8] Fisk JE, Sharp CA (2004) Age-related impairment in executive functioning: Updating, inhibition, shifting, and access. J Clin Exp Neuropsychol 26: 874–890. 15742539 10.1080/13803390490510680

[9] Lehto JE, Juujarvi P, Kooistra L, Pulkkinen L (2003) Dimensions of executive functioning: Evidence from children. Br J Dev Psychol 21: 59–80.

[10] Miyake A, Friedman N, Emerson M, Witzky A, Howerter A, et al (2000) The unity and diversity of executive functions and their contributions to complex "Frontal Lobe" task : a latent variable analysis. Cogn Psychol 41: 49–100. 10945922 10.1006/cogp.1999.0734

[11] Verdejo-García A, Pérez-García M (2007) Profile of executive deficits in cocaine and heroin polysubstance users: common and differential effects on separate executive components. Psychopharmacology 190: 517–530. 17136401 10.1007/s00213-006-0632-8

[12] Barnett JH, Sahakian BJ, Werners U, Hill KE, Brazil R, et al (2005) Visuospatial learning and executive function are independently impaired in first episode psychosis. Psychol Med 35: 1031–1041. 16045069 10.1017/s0033291704004301

[13] Bömmer I, Brüne M (2006) Social cognition in "pure" delusional disorder. Cogn Neuropsychiatry 11: 493–503. 17354084 10.1080/13546800500359994

[14] Bömmer I, Brüne M (2007) Neuropsychological aspects of delusional disorders. Characteristic attributional style or cognitive deficit? Nervenarzt 78: 796–801. 17123122 10.1007/s00115-006-2213-9

[15] Conway CR, Bollini AM, Graham BG, Keefe RS, Schiffman SS, et al (2002) Sensory acuity and reasoning in delusional disorder. Compr Psychiatry 43: 175–178. 11994833 10.1053/comp.2002.32358

[16] Evans JD, Paulsen JS, Harris MJ, Heaton RK, Jeste DV (1996) A clinical and neuropsychological comparison of delusional disorder and schizophrenia. J Neuropsychiatry Clin Neurosci 8: 281–286. 8854299 10.1176/jnp.8.3.281

[17] Fujii DE, Ahmed I, Takeshita J (1999) Neuropsychologic implications in erotomania: two case studies. Neuropsychiatry Neuropsychol Behav Neurol 12: 110–116. 10223258

[18] Herlitz A, Forsell Y (1996) Episodic memory deficit in elderly adults with suspected delusional disorder. Acta Psychiatr Scand 93: 355–361. 8792905 10.1111/j.1600-0447.1996.tb10660.x

[19] Jibiki I, Kagara Y, Kishizawa S, Kurokawa K, Fukushima T, et al (1994) Case-Study of Monosymptomatic Delusion of Unpleasant Body Odor with Structural Frontal Abnormality. Neuropsychobiology 30: 7–10. 7969861 10.1159/000119127

[20] Lapcin S, Guler J, Ceylan E, Maner F, Ger C, Satmis N (2008) Attention impairment in patients with paranoid schizophrenia and delusional disorder: A controlled study. Int J Neuropsychopharmacol 11: 251.

[21] Lapcin S, Guler J, Maner F, Ceylan E, Keskinkilic C, et al (2008) Verbal learning and verbal memory deficits in patients with paranoid schizophrenia and delusional disorder: A controlled study. Int J Neuropsychopharmacol 11: 251.

[22] Leposavic I, Leposavic L, Jasovic-Gasic M (2009) Neuropsychological Profile of Delusional Disorder. Psychiatr Danub 21: 166–173. 19556944

[23] van Os J (2003) Is there a continuum of psychotic experiences in the general population? Epidemiol Psichiatr Soc 12: 242–252. 14968483 10.1017/s1121189x00003067

[24] De Portugal E, Gonzalez N, Vilaplana M, Haro JM, Usall J, et al (2009) Un estudio empirico de los correlatos psicosociales y clinicos del trastorno delirante: el estudio DELIREMP. Rev Psiquiatr Salud Ment 2: 72–82. 23034241 10.1016/S1888-9891(09)72248-7

[25] First MB, Spitzer RL, Gibbon M, Williams JBW (2001) Version Española de la Entrevista Clínica Estructurada para los Trastornos del Eje I del DSM-IV. Barcelona: Masson.

[26] Sheehan DV, Lecrubier Y, Sheehan KH, Amorim P, Janavs J, et al (1998) The Mini-International Neuropsychiatric Interview (M.I.N.I.): the development and validation of a structured diagnostic psychiatric interview for DSM-IV and ICD-10. J Clin Psychiatry 59: 22–33. 9881538

[27] Barona A, Reynolds CR, Chastian RA (1984) Demographically based index of premorbid intelligence for the WAIS-R. J Clin Neuropsychol 8: 169–173.

[28] Bilbao-Bilbao A, Seisdedos N (2004) Eficacia de una fórmula de estimación de la inteligencia premórbida en población española. Rev Neurol 38: 431–434. 15029520

[29] Army Individual Test Battery (1944) Manual of directions and scoring. Washington, DC: War Department, Adjutant Generaĺs Office.

[30] Lezak M. (2004) Neuropsychological assessment (4th ed.). New York: Oxford University Press.

[31] Moll J, de Oliveira-souza R, Moll FT, Bramati IE, Andreiuolo PA (2002) The cerebral correlates of set-shifting - An fMRI study of the Trail Making Test. Arq Neuro-Psiquiatr 60: 900–905. 10.1590/s0004-282x2002000600002 12563376

[32] Mraz R, Zakzanis KK, Graham SJ (2011) An FMRI study investigating the neural substrates for cognitive set-shifting during the Trail Making Test. Arch Clin Neuropsychol 19: 905–906.

[33] Heaton, R K. (2000) WCST-64:CV Computer Version for Windows Research Edition. Odessa, FL: Psychological Assessment Resources.

[34] Steinmetz JP, Houssemand C (2011) What about Inhibition in the Wisconsin Card Sorting Test? Clin Neuropsychol 25: 652–669. 21547856 10.1080/13854046.2011.568525

[35] Simon AE, Giacomini V, Ferrero F, Mohr S (2003) Is executive function associated with symptom severity in schizophrenia? European Archives of Psychiatry and Clinical Neuroscience 253: 216–218. 12910354 10.1007/s00406-003-0421-x

[36] Golden C (1994) Stroop Test de Colores y Palabras: Manual. Madrid: Publicaciones de psicología aplicada.

[37] Dimoska-Di Marco A, McDonald S, Kelly M, Tate R, Johnstone S (2011) A meta-analysis of response inhibition and Stroop interference control deficits in adults with traumatic brain injury (TBI). J Clin Exp Neuropsychol 33: 471–485. 21229434 10.1080/13803395.2010.533158

[38] Heaton RK (1981) A manual for the Wisconsin Car Sorting Test. Odessa, FL: Psychological Assessment Resources.

[39] Benedet M J, Alejandre MA (1998) Manual del Test de Aprendizaje Verbal España Complutense (TAVEC). Madrid: Ediciones TEA.

[40] Delis DC, Kramer JH, Kaplan E, Ober BA (1987) California Verbal Learning Test: Adult version manual. Research Edition. San Antonio, TX: The Psychological Corporation.

[41] Delis DC, Freeland J, Kramer JH, Kaplan E (1988) Integrating clinical assessment with cognitive neuroscience: Construct validation of the California Verbal Learning Test. J Consult Clin Psychol 56: 123–130. 3346437 10.1037//0022-006x.56.1.123

[42] Kraepelin E (1896) Lehrbuch der Psychiatrie, 5th edn. Leipzig: Barth.

[43] Walston F, David AS, Charlton BG (1998) Sex differences in the content of persecutory delusions: A reflection of hostile threats in the ancestral environment? Evol Hum Behav 19: 257–260.

[44] Larrabee GJ, Curtiss G (1995) Construct validity of various verbal and visual memory tests. J Clin Exp Neuropsychol 17: 536–547. 7593474 10.1080/01688639508405144

[45] Mazurek I, Bartyzel M, Loza B, Mosiolek A, Opielak G (2004) P.2.142 Neurocognitive measures of prefrontal cortical dysfunction in schizophrenia: Prefrontal fast oscillations and WCST in patients with schizophrenia. Eur Neuropsychopharmacol 14: S295.

[46] Fear C, Sharp H, Healy D (1996) Cognitive processes in delusional disorders. Br J Psychiatry 168: 61–67. 8770430 10.1192/bjp.168.1.61

[47] Groth-Marnat G (2000) Introduction to neuropsychological assessment. In: Gary Groth-Marnat, editors. Neuropsychological assessment in clinical practice. New York: Wiley.

[48] Garety PA, Freeman D (1999) Cognitive approaches to delusions: a critical review of theories and evidence. Br J Clin Psychol 38: 113–154. 10389596 10.1348/014466599162700

[49] Freeman D, Gittins M, Pugh K, Antley A, Slater M, et al (2008) What makes one person paranoid and another person anxious? The differential prediction of social anxiety and persecutory ideation in an experimental situation. Psychol Med 38: 1121–1132. 18533055 10.1017/S0033291708003589 PMC2830058

[50] Villalta-Gil V, Vilaplana M, Ochoa S, Haro JM, Dolz M, et al (2006) Neurocognitive performance and negative symptoms: are they equal in explaining disability in schizophrenia outpatients? Schizophr Res 87: 246–253. 16859898 10.1016/j.schres.2006.06.013

